# Horizontal transfer of β-carbonic anhydrase genes from prokaryotes to protozoans, insects, and nematodes

**DOI:** 10.1186/s13071-016-1415-7

**Published:** 2016-03-16

**Authors:** Reza Zolfaghari Emameh, Harlan R. Barker, Martti E. E. Tolvanen, Seppo Parkkila, Vesa P. Hytönen

**Affiliations:** School of Medicine, University of Tampere, Medisiinarinkatu 3, FI-33520 Tampere, Finland; BioMediTech, University of Tampere, FI-33520 Tampere, Finland; Fimlab Laboratories Ltd and Tampere University Hospital, FI-33520 Tampere, Finland; Department of Information Technology, University of Turku, FI-20520 Turku, Finland

**Keywords:** Horizontal gene transfer, Mobile genetic elements, Plasmid, Beta carbonic anhydrase, Transposase, Integrase, Resolvase, Endosymbionts, Parasite, Evolution

## Abstract

**Background:**

Horizontal gene transfer (HGT) is a movement of genetic information occurring outside of normal mating activities. It is especially common between prokaryotic endosymbionts and their protozoan, insect, and nematode hosts. Although beta carbonic anhydrase (β-CA) plays a crucial role in metabolic functions of many living organisms, the origin of *β-CA* genes in eukaryotic species remains unclear.

**Methods:**

This study was conducted using phylogenetics, prediction of subcellular localization, and identification of β-CA, transposase, integrase, and resolvase genes on the MGEs of bacteria. We also structurally analyzed β-CAs from protozoans, insects, and nematodes and their putative prokaryotic common ancestors, by homology modelling.

**Results:**

Our investigations of a number of target genomes revealed that genes coding for transposase, integrase, resolvase, and conjugation complex proteins have been integrated with *β-CA* gene sequences on mobile genetic elements (MGEs) which have facilitated the mobility of *β-CA* genes from bacteria to protozoan, insect, and nematode species. The prokaryotic origin of protozoan, insect, and nematode β-CA enzymes is supported by phylogenetic analyses, prediction of subcellular localization, and homology modelling.

**Conclusion:**

MGEs form a complete set of enzymatic tools, which are relevant to HGT of *β-CA* gene sequences from prokaryotes to protozoans, insects, and nematodes.

**Electronic supplementary material:**

The online version of this article (doi:10.1186/s13071-016-1415-7) contains supplementary material, which is available to authorized users.

## Background

Horizontal, or lateral, gene transfer (HGT or LGT) refers to movement of genetic information across normal mating barriers, between more or less phylogenetically distinct organisms, and thus stands in distinction to the standard vertical transmission of genes from parent to offspring. HGT is proving to be a more influential evolutionary mechanism than 20th-century scientists ever thought [1]. Most early, and even current, evidence for HGT in eukaryotes comes from study of protists [2, 3].

Mobile genetic elements (MGEs) are segments of DNA, encoding enzymes and other proteins, which mediate the movement of DNA in HGT within genomes (intracellular mobility) or between cells (intercellular mobility) [4]. Transposases and site-specific recombinases catalyse the intracellular movement of MGEs. Site-specific recombinases in bacteria fall into one of two very distinct families, the λ integrase-like enzymes and the resolvases/invertases [5]. Recombinase interacts with a specific site in the DNA, brings the sites together in a synapse, and religates exchanged DNA strand to the host genome. Homologous recombination systems of the host also enable them to function in chromosomal deletions and other rearrangements [6]. The majority of horizontally transferred genes are either eventually excluded or rapidly become nonfunctional in the recipient genome. However, there are some reports where horizontally transferred genes have shown high level of transcription [6, 7].

Many protists are phagotrophic and subsist by consuming bacteria. Subsequently, protozoan phagotrophs often live for long periods in environments where they are frequently exposed to bacterial DNA. One such example is the direct contact of bacteria and parasites in digestive system of ruminants [2].

In addition, previous literature has demonstrated numerous well-established endosymbiotic partnerships between a variety of eukaryotic hosts and prokaryotic or eukaryotic endosymbionts [8–19]. The close inter-organismal interaction between the host and endosymbiont also provides an opportunity for HGT. Two prominent endosymbiotic relationships in eukaryotic evolution resulted in adoption of mitochondria and plastids from *α-proteobacteria* and *cyanobacteria* species, respectively. Among *Eubacteria*, HGT is involved in the evolution of antibiotic resistance, pathogenicity, and metabolic pathways [20]. Both endosymbiotic and pathogenic prokaryotes are usually considered as the HGT DNA donors to protozoans, insects, and nematodes [21] (Table 1).

**Table 1:** Examples of HGT of prokaryotic genes to protozoans, insects, and nematodes

Prokaryotic gene donors	Protozoan, insect and nematode gene recipients	Horizontally transfered genes
*Wolbachia*	*Aedes aegypti* (yellow fever mosquito), *Anopheles gambiae* (malaria mosquito), and *Drosophila melanogaster*	Many prokaryotic genes, such as gag-pol, D34 immunodominant antigen, actin and aminotransferase genes [65, 66]
*Escherichia coli*	*Caenorhabditis elegans*	Antibiotic-resistance genes [67]
Prokaryotes	Anaerobic protozoans: *Trichomonas vaginalis*, *Entamoeba histolytica,* and *Naegleria gruberi*	Alcohol dehydrogenase (*adh* gene) and *Pyruvate:ferredoxin oxidoreductase* genes [1, 68]
Prokaryotes	*Dictyostelium discoideum* (soil-living amoeba)	18 prokaryotic genes [48]
Prokaryotes	Trypanosomatids: *Leishmania* spp., *Angomonas deanei* and *Strigomonas culicis*	Bacterial amino acid pathways [58]
*α-proteobacteria*	*Leishmania* spp.	Mitochondria (initiation point of apoptosis) [69]
*β-proteobacteria* and *γ-proteobacteria*	Trypanosomatids: *Leishmania* spp., *Angomonas deanei* and *Strigomonas culicis*	*Heme synthesis* gene [50]
*Peptostreptococcus harei*	*Trichomonas vaginalis*	Lateral gene transfer fragment (TvLF) [51]

Carbonic anhydrases (CAs) are ubiquitous metalloenzymes, which belong to six evolutionary divergent gene families, including α, β, γ, δ, ζ, and η [22, 23]. The active site of most CAs contains a zinc ion (Zn^2+^) which plays a critical role in the catalytic activity of the enzyme. CAs are involved in many biological processes, such as respiration involving transport of CO_2_ and bicarbonate between metabolizing tissues, regulation of pH homeostasis, electrolyte transfer, bone resorption, calcification, tumor progression, gluconeogenesis, lipogenesis, and ureagenesis [24–27]. In the past decade, a large number of putative β-CAs have been discovered in protozoans, arthropods, and nematodes [28–32], as well as in bacteria, fungi, algae, and plants [33]. Despite the presence of β-CA sequences in genomes of many, if not most, living organisms, they are absent in vertebrate genomes [28, 29].

In this study, we investigated the possible origin of *β-CA* gene sequences in protozoans, insects, and nematodes by HGT from ancestral prokaryotes using phylogenetics, prediction of subcellular localization, and identification of β-CA, transposase, integrase, and resolvase genes on the MGEs of bacteria. We also structurally analyzed β-CAs from protozoans, insects, and nematodes and their putative prokaryotic common ancestors, by homology modelling. Our study suggests that HGT likely explains the presence of similar *β-CA* genes across multiple species living together in distinct environments.

## Methods

### Identification of *β-CA* gene and protein sequences

We collected all β-CA protein expressing bacteria which are endosymbiotic or pathogenic to a protozoan, insect, or nematode species from Uniprot (http://www.uniprot.org/) and EMBL-EBI databases (http://www.ebi.ac.uk/) (Additional file 1). In addition, we included ten β-CA protein sequences from endosymbiotic bacteria of protozoans, insects, and nematodes to the identification process, including: *Afipia* spp. (K8NQ88), *Anaeromyxobacter* spp. (A7HD59), *Campylobacter* spp. (K0I0K3), *Salmonella* spp. (Q8ZRS0), *Gardnerella* spp. (E3D7T4), *Emticicia* spp. (I2EZ21), *Simkania* spp. (F8L9G5), *Nostoc* spp. (Q8YT17), *Exiguobacterium* spp. (K0ACL8), and *Fusobacterium* spp. (C6JPI1). Moreover, we performed protein homology BLAST search for β-CA protein sequences from protozoans, insects, and nematodes in the EMBL-EBI BLAST database (http://www.ebi.ac.uk/Tools/sss/fasta/) to define bacterial β-CA protein homologs. A highly conserved region (102 amino acid residues, starting from three amino acid residues prior to the first highly conserved motif (CXDXR) was extracted from bacterial, protozoan, insect, and nematode β-CA protein sequences. These sequences were aligned using the Clustal Omega multiple sequence alignment (MSA) algorithm (http://www.ebi.ac.uk/Tools/msa/clustalo/) [34], and the results were visualized in Jalview (http://www.jalview.org/) [35].

### Phylogenetic analysis

A total of 220 β-CA sequences were retrieved from various databases and sorted into sub-groups (clades) based on identification by the Conserved Domain Database server (http://www.ncbi.nlm.nih.gov/Structure/cdd/wrpsb.cgi) [36]. Phylogenetic trees were constructed individually for each β-CA sub-group (clade A-D). The total numbers of sequences analyzed for each sub-group were 109(A), 53(B), 36(C), and 22(D). Four incomplete sequences were corrected, including three from *Naegleria gruberi,* which replace UniProt entries D2W4H2, D2W1R2, and D2W492, and one from *Leishmania braziliensis*, which replaces UniProt entry A4H4M7. In these corrections, the target species genome was analyzed by the Exonerate program [37], using complete β-CA sequences as queries, followed by a comparative analysis of a Clustal Omega alignment of the predictions [34]. For each of the clades A to D, the final set of protein sequences was aligned using Clustal Omega, and a corresponding alignment of coding sequences (CDS) was created by Pal2Nal [38]. Each set of sequences were analyzed using supercomputer resources provided by the Finnish IT Center for Science. The first method applied was Bayesian inference within the MrBayes v3.2.3 program [39], using the General Time Reversible (GTR) nucleotide model until the standard deviation of split frequencies was <0.01. A second analysis by maximum likelihood was completed using PhyML with 1000 bootstrap replicates [40] (Table 2).

**Table 2:** Predicted sources of the β-CA genes. The tentative prokaryotic endosymbionts and their hosts are listed

β-CA clades	Tentative prokaryotic endosymbiont (donor)	Bacterial group	Protozoan, insect, and nematode hosts (acceptor)
A	*Cesiribacter andamanensis* (M7MX87)	Bacteroidetes	*Acanthamoeba castellanii* (L8GR38) (Fig. 2a)
A	*Leptospira kirschneri* (M6X652)	Spirochaetes	*Naegleria gruberi* (Predicted 1, 2, 3)
			*Paramecium tetraurelia* (A0BD61, A0CEX6, A0C922, A0BDB1, A0E8I0) (Fig. 2a)
A	*Colwellia psychrerythraea* (Q47YG3)	Gammaproteobacteria	*Ichthyophthirius multifiliis* (G0QYZ1, G0QPN9)
			*Tetrahymena thermophila* (Q22U21, Q22U16, I7M0M0, I7M748, I7LWM1, I7MDL7, Q23AV1, I7MD92)
			*Dictyostelium* spp (Q555A3, Q55BU2, Q94473, F0Z7L1, F4PL43) (Fig. 2a)
A	*Magnetospirillum magneticum* (Q2VZD0)	Alphaproteobacteria	*Angomonas daenei* (S9WXX9)
			*Strigominas culicis* (S9TM82)
			*Leishmania* spp (A4H4M7 as predicted, E9B8S3, A4HSV2, Q4QJ17, E9AKU0, S0CTX5) (Fig. 2a)
B	*Myxococcales*	Deltaproteobacteria	Insects and nematodes (F1LE18, G4V6B2, Q22460, Q5TU56, Q17N64, Q9VHJ5) (Fig. 2b)
C	*Vesicomyosocius okutanii* (A5CVM8)	Gammaproteobacteria	*Entamoeba* spp (B0E7M0, 1C4LXK3, K2GQM0) (Fig. 2c)
C	*Afipia felis* (K8NQ88)	Alphaproteobacteria	*Acanthamoeba castellanii* (L8GLS7) (Fig. 2c)
	*Bradyrhizobium japonicum* (G7D846)		
D	*Selenomonas ruminantium* (I0GLW8)	Firmicutes	*Trichomonas vaginalis* (A2ENQ8, A2DLG4) (Fig. 2d)
	*Veillonella* spp (F9N508)		

### Prediction of subcellular signals

Prediction of subcellular signals of defined protozoan, insect, and nematode β-CA protein sequences was performed using a subcellular signal prediction tool. Mitochondrial and secretory targeting peptides in β-CA protein sequences were predicted by TargetP 1.1 Server (http://www.cbs.dtu.dk/services/TargetP/) [41]. Even if these targeting systems are only found in eukaryotes, bacterial sequences were analyzed as well to see if they contain regions similar to eukaryotic targeting signals. Based on the phylogenetic tree results, we performed this analysis on only those bacterial β-CA protein sequences, which had a predicted common ancestor with protozoan, insect, or nematode β-CA protein sequences. Specifically, this included *Afipia felis* (K8NQ88), *Bradyrhizobium japonicum* (G7D846), *Cesiribacter andamanensis* (M7MX87), *Colwellia psychrerythraea* (Q47YG3), *Corallococcus coralloides* (H8MJ17), *Leptospira kirschneri* (M6X652), *Magnetospirillum magneticum* (Q2VZD0), *Selenomonas ruminantium* (I0GLW8), *Veillonella* spp. (F9N508), and *Vesicomyosocius okutanii* (A5CVM8).

### Identification of β-CA, transposase, integrase, resolvase, and conjugation complex protein (CCP) genes on the prokaryotic MGEs

Identification of β-CA, transposase, integrase, resolvase, and CCP genes on the bacterial MGEs was carried out using the plasmid database from EMBL-EBI (http://www.ebi.ac.uk/genomes/plasmid.html), and the Jena Prokaryote Genome Viewer (JPGV) (http://jpgv.fli-leibniz.de/cgi/index.pl) [42]. JPGV contains a vast amount of information on most fully sequenced prokaryotic genomes and presents figures of linear and circular genome plots.

### Identification of *β-CA* gene sequences on protozoan, insect, and nematode genomic DNA

Analyses regarding determination of precise locations of protozoan, insect and nematode *β-CA* genes in genomic DNA were performed using National Center for Biotechnology Information (NCBI) database (http://www.ncbi.nlm.nih.gov/). Furthermore, we utilized the *Trichomonas vaginalis* genome project database (TrichDB version 1.3) (http://trichdb.org/trichdb/) [43] and EMBL-EBI database (http://www.ebi.ac.uk/), for detection of *β-CA* genes in *Trichomonas vaginalis* (a protozoan parasite and the causative agent of trichomoniasis) and *C. elegans* respectively. Analysis of mitochondrial coding genes in *Acanthamoeba castellanii* (the most common free-living amoeba in soil and water) was performed using the NCBI database (http://www.ncbi.nlm.nih.gov/).

### Homology modelling

Homology models were prepared for β-CAs selected based on the phylogenetic analysis. The most similar eukaryotic and prokaryotic proteins within the phylogeny tree branch in question were selected using the percent identity matrix generated by Clustal Omega (http://www.ebi.ac.uk/Tools/msa/clustalo/) [34]. For each of the selected proteins, the most similar protein structure was obtained using BLAST search targeted for the PDB database (http://www.rcsb.org/pdb/home/home.do). For each protein pair (eukaryotic and prokaryotic) analyzed here, the BLAST search resulted in the same template protein as follows: Clade A: *Escherichia coli* β-CA PDB 1I6P; Clade B: *Pisum sativum* β-CA PDB 1EKJ; Clade C: *Mycobacterium tuberculosis* β-CA PDB 1YM3; and Clade D: *Methanobacterium thermoautotrophicum* β-CA PDB 1G5C.

Clustal Omega (http://www.ebi.ac.uk/Tools/msa/clustalo/) [34] was used to prepare a sequence alignment for the modelled protein and the template protein sequence. The homology models were prepared using Modeller program (version 9.14) [44]. The resulting models were structurally aligned by the BODIL program [45]. A figure illustrating the homology models was prepared using the VMD program (version 1.9.1) [46] and edited with Adobe Photoshop (version 13.0.1).

The evaluation of the conserved residues in the homology models was performed by using multiple sequence alignments prepared by Clustal Omega algorithm (http://www.ebi.ac.uk/Tools/msa/clustalo/) [34] and by inspecting the homology models using program VMD program (version 1.9.1) [46].

## Results

### Identification and phylogenetic analysis of β-CA protein sequences from defined bacterial, protozoan, insect, and nematode species

**Fig. 1:**
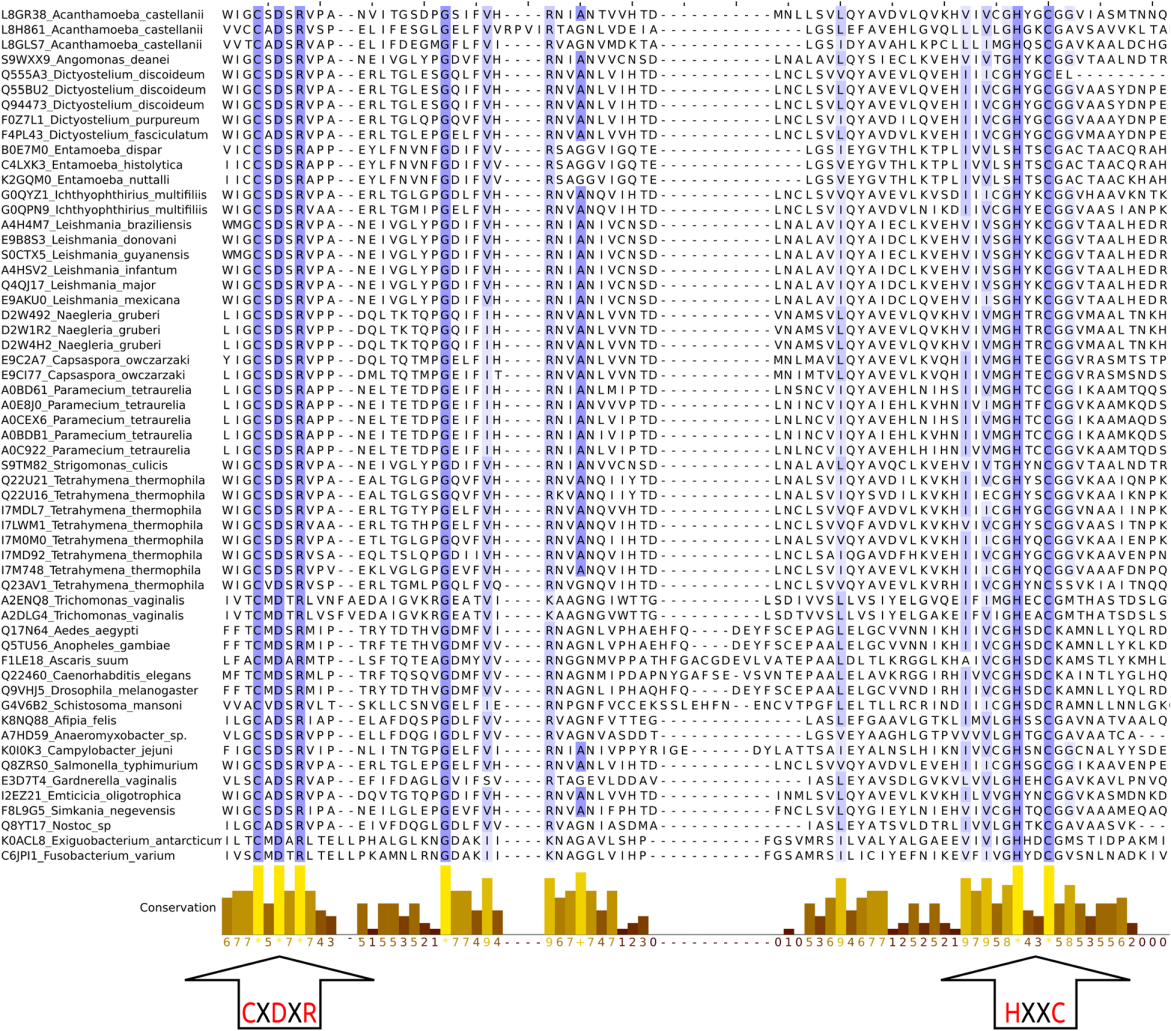
Multiple sequence alignment (MSA) of 57 β-CA protein sequences. They include sequences (102 amino acid residues starting three amino acid residues prior to the first highly conserved sequence; CXDXR) from defined protozoan, insect, and nematode species, as well as ten β-CA protein sequences from bacterial endosymbionts of protozoans, insects, and nematodes, and *Afipia* spp. (K8NQ88), *Anaeromyxobacter* spp. (A7HD59), *Campylobacter* spp. (K0I0K3), *Salmonella* spp. (Q8ZRS0), *Gardnerella* spp. (E3D7T4), *Emticicia* spp. (I2EZ21), *Simkania* spp. (F8L9G5), *Nostoc* spp. (Q8YT17), *Exiguobacterium* spp. (K0ACL8), and *Fusobacterium* spp. (C6JPI1). First (CXDXR) and second (HXXC) highly conserved motifs of β-CAs are shown with two black arrows at the bottom of the figure

Multiple sequence alignment (MSA) of β-CA protein sequences from protozoan, insect, nematode species with bacterial β-CA protein sequences, revealed that all the aligned sequences included both the first (CXDXR; C: Cysteine, D: Aspartic acid, R: Arginine, and X: any residue) and second (HXXC; H: Histidine, C: Cysteine, X: any residue) highly conserved motifs of the active site (Fig. 1).

**Fig. 2:**
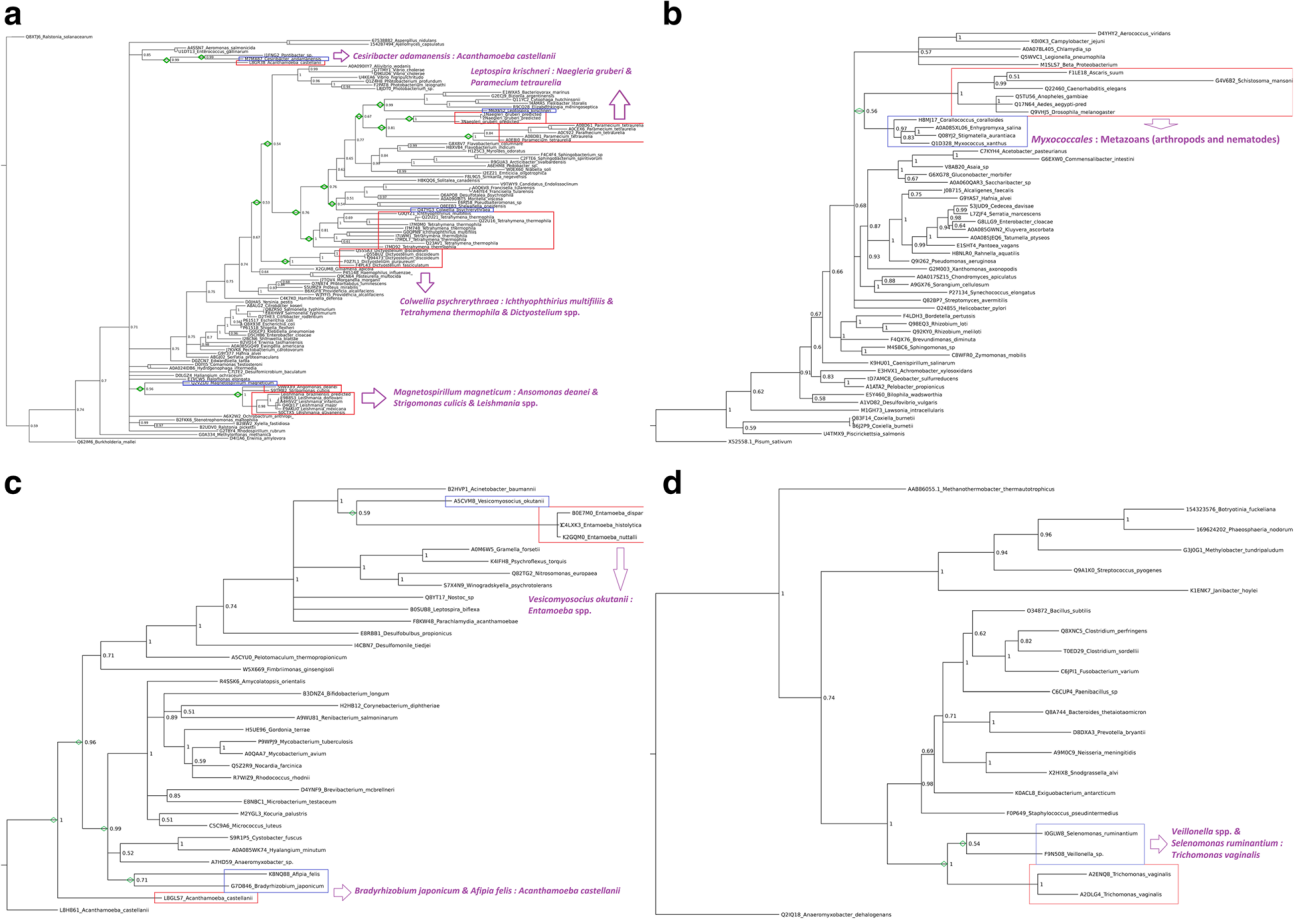
Phylogenetic analysis of clade **a**, **b**, **c**, and **d** of β-CA protein sequences. Eukaryotic hosts and tentative prokaryotic endosymbionts are pinpointed in red and blue boxes, respectively. The green diamonds at internal nodes represent common ancestors which have both bacterial and eukaryotic descendants, and identify the possible pathways of β-CA HGT from common bacterial sources to protozoan, insect, and nematode species. The plausible HGT of *β-CA* genes from tentative prokaryotic endosymbionts to eukaryotic hosts are shown by purple arrows and by indicating names of the donor and acceptor species

Phylogenetic analyses of clade A, B, C, and D of β-CA protein sequences revealed the common ancestor of protozoan, insect, and nematode β-CAs within bacterial β-CA protein sequences (Fig. 2a-d) (Table 3).

**Table 3:** MrBayes/PhyML Settings and Results of Phylogentic Analysis

β-CA Clade	Sequences	MrBayes	MrBayes	MrBayes	PhyML Boot Straps
		Iterations	Std Dev of Split Freq	Trees sampled	
A	109	365,000,000	0.0092	273515	1000
B	53	35,000,000	0.0087	25812	1000
C	36	2,000,000	0.0077	7501	1000
D	22	350,000	0.0055	1314	1000

### Prediction of subcellular signals

Prediction of subcellular signals revealed that five protozoan (L8GR38, A4H4M7, S0CTX5, S9TM82, and I7MDL7) and three insect (Q5TU56, Q17N64, and Q9VHJ5) β-CA proteins probably contain mitochondrial targeting peptides. Even three bacterial β-CA proteins (K8NQ88, H8MJ17, and M6X652) contained N-terminal sequences sufficiently similar to mitochondrial targeting peptides so that mitochondrial prediction by TargetP 1.1 Server was positive. In addition, one protozoan β-CA protein (L8H861) sequence from *A. castellanii* is predicted to contain a signal peptide for the secretory pathway. The prediction tool provided no definitive localization for the other bacterial, protozoan, insect, and nematode β-CA proteins (Additional file 2).

### Identification of β-CA, transposase, integrase, resolvase, and conjugation complex protein (CCP) coding sequences on the bacterial MGEs

**Fig. 3:**
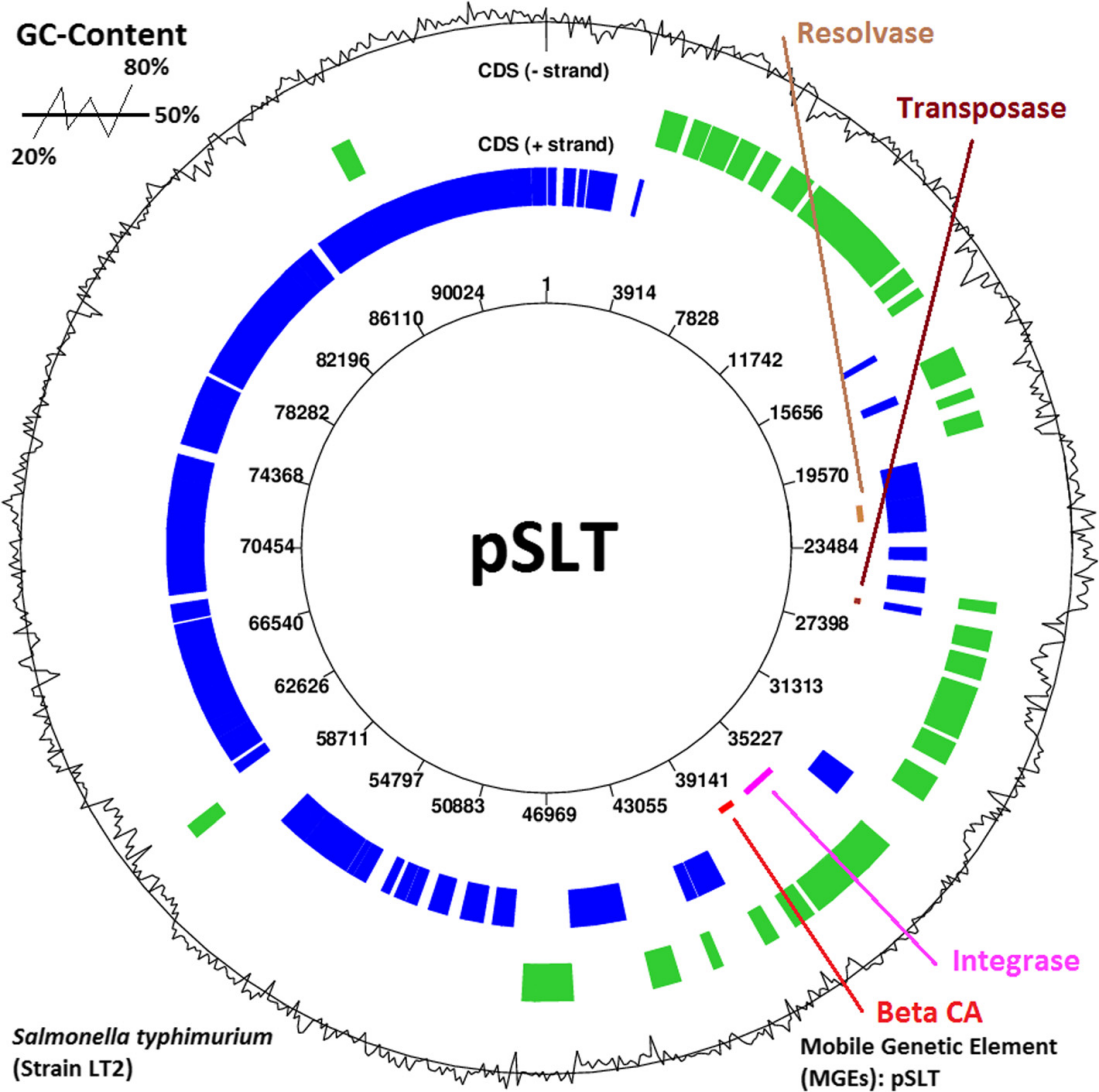
Circular structure of plasmid pSLT from *S. typhimurium*, strain LT2. The mobile genetic element pSLT contains β-CA (37,528-38,268 bp), transposase (25,877-26,140 bp), integrase (35,113-36,777 bp), and resolvase (21,466-22,248 bp) genes. Line graph along outer circumference of MGE model represents G + C content of pSLT, which is lower or higher than baseline (50 %)

In order to study the genomic context of *β-CA* genes and to understand the molecular mechanisms involved in HGT, we explored the association of prokaryotic *β-CA* genes in MGEs. The ACLAME version 0.4 database (http://aclame.ulb.ac.be/) [47] enabled us to first identify a *β-CA* gene within the pSLT mobile genetic element of *Salmonella typhimurium* (str. LT2) (data not shown). Subsequent analysis within other MGE browsers, including EMBL-EBI (http://www.ebi.ac.uk/genomes/plasmid.html) and Jena Prokaryote Genome Viewer (JPGV) (http://jpgv.fli-leibniz.de/cgi/index.pl) databases, led to discovery of 40 *β-CA* genes located within MGEs in different prokaryotic species. Each bacterial MGE contained only one *β-CA* gene sequence and occasionally several transposase, integrase, resolvase, and CCP coding genes. MGEs were found to differ from each other by length, number of coding genes, and encoded proteins. Each β-CA, transposase, integrase, resolvase, and CCPs were identified by specific coding IDs from ACLAME and GenBank and only one instance of each protein is listed (Additional file 3) for each bacterial species as a representative example. The study of ACLAME data shows that β-CA is found in evolutionary conserved modules of MGEs, even at the most stringent significance thresholds. The locations of β-CA, transposase, integrase, and resolvase gene sequences in plasmid pSLT from *S. typhimurium* (strain LT2) are shown in Fig. 3. The figure shows that pSLT expresses transposase, integrase, and resolvase as the main enzymatic tools, which facilitate the HGT of *β-CA* gene in this plasmid and similar configuration was observed in the case of several other MGEs.

### Identification of *β-CA* gene sequences on protozoan, insect, and nematode genomic DNA

Analysis of the precise location of *β-CA* gene sequences in protozoan, insect, and nematode genetic structures revealed that all were located in chromosomal DNA (Additional file 4). Exon counts, for the group of studied *β-CA* gene sequences, vary in quantity from 1 to 11. The maximum exon counts were 8 for *A. castellanii* (Entry ID: L8GR38), and 11 for *P. pacificus* (Entry ID: H3EVA6) for protozoan and nematode species, respectively. Interestingly, some protozoan *β-CA* gene sequences included only one exon. The definitive locations of *β-CA* gene sequences are shown on linear genomic DNA from *T. vaginalis* (A2DLG4) (Additional file 5) and *C. elegans* (Q22460) (Additional file 6), whereas they are still unknown in many species. Analysis of the genes on circular mitochondrial DNA from *A. castellanii* revealed that none of the protozoan *β-CA*s were considered mitochondrial coding genes (data not shown).

### Homology models

**Fig. 4:**
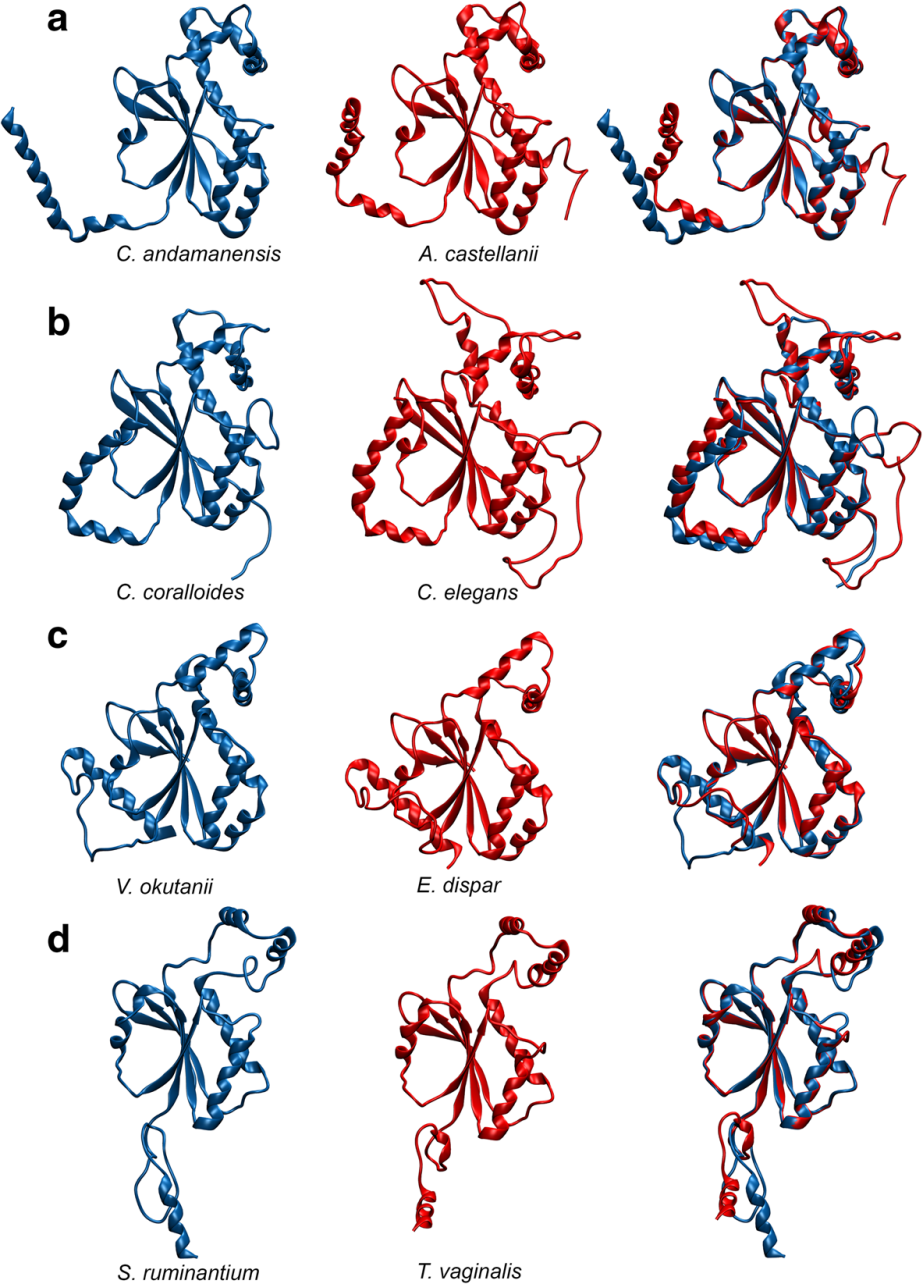
Homology models of representative pairs of β-CAs from clades **a**, **b**, **c**, and **d**. The blue protein models correspond to prokaryotic proteins and the red models to eukaryotic proteins. The superimposed models were shown in the third column at the right side

Homology modelling further supported the idea of high similarity within the inspected protein groups from prokaryotes and protozoans-metazoans (insects and nematodes). No large insertions or deletions were observed and the majority of structural variation is located in the termini of the polypeptide chains. The superimposed homology models created from a pair of proteins from each clade of the β-CAs are shown in Fig. 4.

## Discussion

Throughout their evolution all eukaryotes have been in close contact with bacteria, and while eukaryotrophs are comparatively rare there are numerous identified bacterial endosymbionts which have adapted to intracellular endosymbiosis with protozoan host species [1, 48–52]. In general, HGT of prokaryotic genes to protozoan genomes is probably much more common than *vice versa* [2]. Interestingly, many bacteria are able to tolerate harsh conditions, such as presence of digestive enzymes in phagocytic vesicles, and survive inside protozoan species without any problems. The mechanisms of these efficient endosymbiotic and HGT phenomena are still unknown. There are multiple examples of highly efficient HGT, such as: from *E. coli* to protozoan ciliates, including *T. thermophila* and *T. pyriformis* [53]; from *Klebsiella* spp. to *Salmonella* spp. within the endosymbiotic environment of rumen protozoa of ruminants [54]; and from endosymbiont bacteria to *Leishmania* spp. during bacterial sepsis [55].

Multiple sequence alignment (MSA) of suspected protozoan, insect, and nematode β-CA protein sequences with previously defined bacterial β-CA proteins, revealed that all of the evaluated sequences contained the first (CXDXR) and second (HXXC) highly conserved motifs characteristic of β-CA proteins. Phylogenetic analysis revealed that protozoan, insect, and nematode β-CA protein sequences are mostly categorized as clade A or B β-CA protein structures, respectively.

Based on our phylogenetic analysis, *A. castellanii* possesses two *β-*CA genes, one from clade A and one from clade C. Our results in Fig. 2c, suggest that the *β-CA* gene of *A. castellanii* (L8GLS7) was potentially horizontally transferred from a bacterial species, which probably was a common ancestor of *B. japonicum* (G7D846) and *A. felis* (K8NQ88). In addition, previous studies have shown that *B. japonicum* [56] and *A. felis* [57] are endosymbionts of *A. castellanii*.

Phylogenetic analysis of clade A β-CAs (Fig. 2a), showed that all β-CAs in *N. gruberi* and *P. tetraurelia* protozoa have a common source with the single β-CA from spirochaetes bacteria, *L. kirschneri* (M6X652). Potentially, after HGT of a *β-CA* gene from the common source to these two protozoan hosts, the gene duplicated and created three different *β-CA* genes for *N. gruberi* (Predicted 1, 2, 3) and five for *P. tetraurelia* (A0BD61, A0CEX6, A0C922, A0BDB1, A0E8I0).

Among the various prokaryotic endosymbionts it is proposed that *I. multifiliis*, *T. thermophila*, and *Dictyostelium* spp. potentially have a distant common source with gammaproteobacteria *C. psychrerythraea* (Q47YG3), because there are multiple branch points between *C. psychrerythraea* and the other prokaryotic species. Gene duplication in these protozoans led to multiple copies of *β-CA* in *I. multifiliis* (G0QYZ1, G0QPN9), *T. thermophila* (Q22U21, Q22U16, I7M0M0, I7M748, I7LWM1, I7MDL7, Q23AV1, I7MD92), and *Dictyostelium* spp. (Q555A3, Q55BU2, Q94473, F0Z7L1, F4PL43) [28].

It has been shown earlier that essential amino acid and heme synthesis genes horizontally transferred from endosymbiont alpha, beta, and gammaproteobacteria to *Trypanosomatidea* [9, 50, 58]. Our phylogenetic results (Fig. 2a) revealed that *β-CA* genes in *Trypanosomatidea*, including *Leishmania* spp. (A4H4M7, E9B8S3, A4HSV2, Q4QJ17, E9AKU0, and S0CTX5), *A. daenei* (S9WXX9), and *S. culicis* (S9TM82) have a common source with an alphaproteobacterium similar to *M. magneticum* (Q2VZD0).

The phylogenetic analysis (Fig. 2b) showed that insect and nematode β-CAs belong to clade B and suggests that they may have a common source with myxobacterial β-CAs. The various myxobacteria *Corallococcus, Enhygromyxa, Stigmatella,* and *Myxococcus*, are part of the same subtree that contains insect and nematode β-CAs. However, a larger analysis with more insect, nematode, and plant β-CAs, which also belong to clade B, would be needed to fully resolve the relationships within this clade. Given the apparent distribution within insects and nematodes, in our limited analysis, this HGT would have occurred in the distant past. A single, very old transfer of *β-CA* gene to insects and nematodes would fit with the idea that heritable transfer to sexually reproducing organisms is significantly more difficult. Due to sequence divergence over 800 million years (estimated divergence time between nematodes and arthropods), our phyologenetic trees do not provide conclusive evidence for this, and it is thus possible to speculate that the β-CAs of clade B, which we see in insects and nematodes, have been retained from an ancestral eukaryote. However, it is tempting to assume that β-CAs of all four clades in protozoans, insects, and nematodes would have been derived by HGT from prokaryotes. In this context, we may also note that the HGT of β-CA gene sequences might have involved several mechanisms and genetic elements in addition to MGEs, such as genomic islands (GIs) and insertion sequence (IS) elements.

Phylogenetic analysis of clade C (Fig. 2c) revealed that *β-CA* genes from *Entamoeba* spp. (B0E7M0, 1C4LXK3, K2GQM0) have a common source with the *β-CA* gene of gammaproteobacterium *V. okutanii* (A5CVM8). From this result, we propose that *β-CA* genes horizontally transferred from an ancestral enteric gammaproteobacteria to *Entamoeba* spp. through a symbiotic or pathogenic relationship in the gut of arthropods, nematodes, or animals.

Phylogenetic analysis of clade D (Fig. 2d) revealed that *β-CA* genes in *T. vaginalis* (A2ENQ8, A2DLG4) have a common source with *β-CA* genes from firmicutes bacteria *S. ruminantium* (I0GLW8) and *Veillonella* spp. (F9N508). Previous results have shown that *Clostridium sordellii* and *Veillonella* spp. from firmicutes phylum and *T. vaginalis* have a symbiotic living situation in sexual organs of animals [18, 19], providing the environment in which a transfer of firmicutes bacteria *β-CA* gene sequence into the *T. vaginalis* genome is possible.

Prediction of subcellular signals of β-CA protein sequences revealed that some bacterial species (*A. felis*, *L. kirschneri*, and *C. coralloides*), protozoan species (*A. castellanii*, *L. braziliensis*, *L. guyanensis*, *S. culicis*, and *T. thermophila*), and insect species (*A. gambiae*, *A. aegypti* and *D. melanogaster*) include mitochondrial signals or similar bacterial sequences in their β-CA protein sequences (Additional file 2). It is well established that prokaryotes and some anaerobic protozoa, such as *G. lamblia*, *E. histolytica, T. vaginalis*, *C. parvum*, *Blastocystis hominis*, *Encephalitozoon cuniculi*, *Sawyeria marylandensis*, *Neocallimastix patriciarum*, and *Mastigamoeba balamuthi* completely lack mitochondria*.* In anaerobic protozoan species, mitochondrion-related organelles (MROs, mitosoms, or hydrogenosomes) replaced mitochondria in oxygen-restricted environments. Many studies have hypothesized that a majority of the mitochondrial genes in anaerobic parasitic protozoa have been acquired from α-proteobacterial genomes [59]. The *Monoamine oxidase* (a mitochondrial outer membrane enzyme for metabolism of neuromediators) gene is one such example, and its sequence has been investigated thoroughly from bacterial to vertebrate lineages [60]. Therefore, we hypothesize that sequences similar to mitochondrial localization signals emerged in β-CA proteins in prokaryotes, leading to their mitochondrial localization after HGT into protozoans and possibly insects. Supporting this idea, the β-CA of *D. melanogaster* has been experimentally shown to be localized in mitochondria [28, 29].

Identification of β-CA with transposase, integrase, resolvase, and CCP coding sequences in bacterial MGEs suggests that these genetic elements are a complete set of enzymatic tools, which are relevant to HGT. These accessory enzymes detect target sites on the genome of recipient protozoan species using complex mechanisms and create a conducive environment for integration of *β-CA* gene sequences. On the other hand, in some MGEs, including pSLT, pOU1113, pSCV50, and pKDSC50 from *S. typhimurium* (str. LT2), *S. enterica*, *S. enterica* (serovar *Choleraesuis*, str. SC-B67), and *S. enterica* (serovar *Choleraesuis*), respectively, β-CA is a virulence factor which is located at 5´ end of the *resolvase* gene [61]. The MGEs from *E. histolytica* contain the coding sequence for B2 DNA polymerase [62]. Analysis of the full genomes of protozoans revealed that all *β-CA* gene sequences were located on a single chromosome, although the precise chromosomal location for some protozoan *β-CA* genes is still pending (Additional file 4).

In order to evaluate the structural features of the identified β-CA proteins, we first analyzed the functional roles of the conserved residues. β-CAs have only a limited number of conserved residues essential for the protein fold and function [63]. We demonstrated this by creating a MSA of the β-CAs included in the homology modelling analysis. Indeed, this analysis indicated strict conservation of only the active site residues plus one glycine (Fig. 1). We then further analyzed the residues conserved in β-CAs where eukaryotic and prokaryotic versions grouped together in phylogenetic analysis, i.e. those that we suspected were the result of HGT. One would expect that high similarity between proteins in distantly related species would exist due to two reasons: (1) convergent evolution or (2) HGT. In the possible case of convergent evolution, there should be a selective pressure towards a particular structural or functional feature in certain locations of the protein sequence. We analyzed this by selecting residues, which were found to be conserved between each pairing of phylogenetically grouped eukaryotic and prokaryotic β-CAs, but not in the β-CAs used as a template in homology modelling. Because this excludes the well known functional active site residues, the remaining conserved residues (especially the side chains) should have a particularly important role in the protein structure to cause convergent evolution. Within the ten conserved residues from the protein core selected for analysis of each homology model, we typically observed only a few hydrophobic contacts and in particular polar interactions were almost completely missing, even when considering possible rotamers of the surrounding residues. The result of this analysis thus implies that there are no structurally important roles for the majority of the conserved residues common for the protein pairs observed in the phylogeny analysis. This suggests that the proteins share their identical residues due to their origins in a relatively recent identical genetic source (HGT), not because of selection pressure towards the particular residue observed in each position.

Our present findings may shed some light into the question of why *β-CA* gene sequences are completely absent in the genomes of vertebrates. In protozoan and invertebrate metazoans, including insect and nematode species, *β-CA* gene sequences have integrated in nuclear chromosomes through the aid of some enzymatic functions included in MGEs, such as transposase, integrase, and resolvase. These enzymes function as site-specific cutters and snip the DNA of the recipient eukaryotic host. There are some possible reasons for the lack of HGT of *β-CA* gene sequences in vertebrate genomes. First, there may not be a specific transposable element insertion site within vertebrate genomes for these enzymatic cutters. Second, vertebrates are complex multicellular organisms in which evolutionarily stable integration of *β-CA* gene sequences would need to have taken place in the germ cells that give rise to egg and sperm cells [64]. Finally, supposing successful integration of a *β-CA* gene sequence in the germ line, it may have then been removed by genetic assortment of the vertebrate hosts. Therefore, the lack of *β-CA* gene sequences from the vertebrate genomes is understandable, especially because there is no evolutionary pressure for the adoption of another CA class due to the presence of several efficient α-CAs in all vertebrates.

## Conclusions

Many prokaryotic MGEs contain necessary enzyme gene sequences, such as transposase, integrase, and resolvase, together with β-CA. These enzymes can facilitate HGT of *β-CA* genes from prokaryotes to other prokaryotes (Pro-Pro) and eukaryotes (Pro-Euk). The results from both mitochondrial targeting signal prediction and phylogenetic analysis supported our hypothesis of HGT of *β-CA* gene sequences from endosymbiont bacteria to protozoan, insect, and nematode hosts by MGEs. The phylogenetic analysis suggests that different protozoan *β-CA* genes have various common ancestors among prokaryotes, divided between clades A, C and D of β-CAs. In contrast, the case of insect and nematode *β-CA* genes is more complex. We propose that they may have had a single common ancestor from a bacterial *β-CA* gene, however, their descent from an ancient eukaryote origin cannot be ruled out. In analysis of the conserved residues in the homology models of prokaryote/eukaryote pairs, we observed no particularly important structural reason for the high sequence homology. This finding speaks against convergent evolution as a reason for the high similarity between the proteins and supports the idea of HGT as a source of the *β-*CA gene in eukaryotic species.

## Additional files

Additional file 1: β-CA expressing prokaryotes and their endosymbiotic protozoan, insect, and nematodes hosts. (PDF 301 kb) Additional file 2: Prediction of subcellular localization of in vitro-approved prokaryotic endosymbionts and protozoan β-CA protein sequences. (PDF 394 kb) Additional file 3: Bacterial MGEs containing β-CA, transposase, integrase, resolvase, and CCP coding sequences. (PDF 338 kb) Additional file 4: Genomic location of *β-CA* gene sequences from protozoan, insect, and nematode species. (PDF 363 kb) Additional file 5: Location of *β-CA* gene sequence (TVAG_268150) in *T. vaginalis.* This gene (Entry ID: A2DLG4) has been located on the linear main genomic DNA sequence from 151,119 to 151,673 nt. Analysis revealed that it consists of only one exon (Additional file 4). (TIF 45 kb) Additional file 6: Location of *β-CA* gene sequence (bca-1) in *C. elegans.* This gene (Entry ID: Q22460) has been located on linear main genomic DNA sequence from 23,095 to 25,694 nt. Analysis revealed that it consists of seven exons (Additional file 4). (TIF 132 kb)
