# Supplementary material for: Horizontal transfer of β-carbonic anhydrase genes from prokaryotes to protozoans, insects, and nematodes
Source: Parasit Vectors. 2016 Mar 16;9:152. doi: 10.1186/s13071-016-1415-7 (PMC4793742; doi:10.1186/s13071-016-1415-7)
Supplement: Additional file 1: — β-CA expressing prokaryotes and their endosymbiotic protozoan, insect, and nematodes hosts. (PDF 301 kb) [file 13071_2016_1415_MOESM1_ESM.pdf]

Additional file 1. β-CA expressing prokaryotes and their endosymbiotic protozoan, insect, and nematode hosts.

**Endosymbiont prokaryotic species**	**Protozoan, insect, and nematode species**
Escherichia coli, *Rickettsia prowazekii*, *Rhodospirillum rubrum*, *Vibrio cholera*, *Afipia felis*, *Bradyrhizobium japonicum*, *Ralstonia pickettii*, *Coxiella burnetii*, *Francisella tularensis*, *Pseudomonas aeruginosa*, *Mycobacterium avium*, *Helicobacter pylori*, *Simkania negevensis*, *Parachlamydia acanthamoebae* [1]	*Acanthamoeba castellanii*
Asaia spp*, Serratia* spp*, Klebsiella* spp*, Asaia* spp*, Bacillus* spp*, Enterococcus* spp*, Enterobacter* spp*, Kluyvera* spp*, Pantoea* spp*, Elizabethkingia, Sphingomonas* spp*, Aeromonas culicicola, Spiroplasma* spp*, Brevibacterium* spp.*, Comamonas* spp.*, Flavobacterium* spp*, Acinetobacter lwoffii, Pseudomonas aeruginosa , Streptococcus* spp*, Erwinia* spp*, Providencia* spp*, Elizabethkingia meningoseptican, Ralstonia paucula* [2-4]	*Aedes aegypti*
Asaia spp*, Pseudomonas* spp*, Elizabethkingia anophelis, Enterobacter* spp*, Aeromonas* spp*, Myroides* spp*, Exiguobacterium* spp*, Shewanella* spp*, Enterococcus* spp*, Kocuria* spp*, Microbacterium* spp*, Klebsiella* spp*, Chryseobacterium* spp*, Rhodococcus* spp*, Alcaligenes* spp*, Bordetella* spp*, Rhodococcus* spp*, Escherichia* spp*, Staphylococcus* spp*, Ewingella* spp*, Serratia* spp*, Flavobacterium* spp*, Acinetobacter* spp*, Pantoea* spp*, Aeromonas* spp*, Cedecea* spp*, Klyvera* spp*, Achromobacter* spp*, Bacillus* spp*, Hydrogenophaga* spp*, Cedecea* spp*, Morganella* spp*, Pantoea* spp*, Salmonella* spp*, Brevundiumonas* spp*, Comamonas* spp*, Flavobacterium* spp*, Gluconobacter* spp*, Erwinia* spp*, Klyvera* spp*, Acidovorax* spp*, Anaplasma* spp*, Mycoplasma* spp*, Paenibacillus* spp*,*	*Anopheles gambiae*

Rhodococcus spp, *Thorsellia anophelis, Vibrio* spp, *Janibacter* spp*, Spiroplasma* spp [5, 6]	
Bifidobacterium spp, *Acinetobacter* spp, *Pseudomonas* spp, *Stenotrophompnas* spp, *Pasteurella* spp, *Enterobacteriaceae*: *Burkholderia* spp, *Citrobacter* spp, *Escherichia* spp, *Enterobacter* spp, *Klebsiella* spp, *Salmonella* spp, *Pantoea* spp, *Yersinia* spp [7]	*Ascaris suum*
Bacillus spp, *Aeromonas* spp, *Burkholderia* spp, *Erwinia* spp, *Pseudomonas* spp, *Serratia* spp, *Shewanella* spp, *Agrobacterium* spp, *Staphylococcus* spp, *Entrococcus* spp, *Streptococcus* spp, *Streptomyces* spp, *Yersinia* spp, *Microbacterium* spp, *Escherichia* spp, *Shigella* spp, *Salmonella* spp, *Listeria* spp, *Photorhabdus* spp, *Xenorhabdus* spp, *Legionella* spp, *Mycobacterium* spp, *Vibrio* spp, *Cronobacter* spp, *Lactobacillus* spp, *Pediococcus* spp, *Proteus* spp, *Streptoverticillium* spp [8]	*Caenorhabditis elegans*
Legionella pneumophila, *Mycobacterium avium*, *Salmonella typhimurium*, *Pseudomonas aeruginosa*, *β-Proteobacterium* [9]	*Dictyostelium* spp.
Commensalibacter spp*, Gluconobacter* spp*, Spiroplasma* spp*, Vibrio* spp*, Lactobacillus* spp*, Acetobacter* spp*,* Enterobacteriaceae: *Edwardsiella* spp*, Enterobacter* spp*, Escherichia* spp*, Klebsiella* spp*, Kluyvera* spp*, Leminorella* spp*, Pantoea* spp*, Proteus* spp*, Providencia* spp*, Rahnella* spp*, Serratia* spp*, Shigella* spp*, Tatumella* spp*, Yersinia* spp*, Bacillus* spp*, Staphylococcus* spp*, Pseudomonas* spp [10]	*Drosophila melanogaster*
Burkholderia mallei, Legionella *pneumophila*, *Rickettsia prowazekii* [11]	*Entamoeba* spp.
Sphingobacterium spiritivorum, Flavobacterium columnare, Caenispirillum salinarum, Rickettsia prowazekii, Emticicia	*Ichthyophthirius multifiliis*

oligotrophica [12]	
Legionella pneumophila [1]	*Naegleria gruberi*
Francisella tularensis, *Rickettsia prowazekii* [13]<br>*Chlorella variabilis* (algae) [14]	*Paramecium tetraurelia*
Fibrobacter spp, *Ruminococcus* spp, *Zymomonas* sp [15]	*Schistosoma mansoni*
Escherichia coli, *Chlorella variabilis* (algae) [16, 17]	*Tetrahymena thermophila*
Gardnerella vaginalis, *Prevotella bryantii*, *Clostridium sordellii* [18, 19]	*Trichomonas vaginalis*
Alpha, beta, and gammaproteobacteria [20, 21]	*Trypanosomatidae* (*Leishmania* spp., *Angomonas deanei*, and *Strigomonas culicis*)

References:

1. Greub G, Raoult D. Microorganisms resistant to free-living amoebae. Clin Microbiol Rev. 2004;17(2):413-33.
2. Gusmao DS, Santos AV, Marini DC, Bacci M, Jr., Berbert-Molina MA, Lemos FJ. Culture-dependent and culture-independent characterization of microorganisms associated with Aedes aegypti (Diptera: Culicidae) (L.) and dynamics of bacterial colonization in the midgut. Acta Trop. 2010;115(3):275-81.
3. Gusmao DS, Santos AV, Marini DC, Russo Ede S, Peixoto AM, Bacci Junior M, et al. First isolation of microorganisms from the gut diverticulum of Aedes aegypti (Diptera: Culicidae): new perspectives for an insect-bacteria association. Mem Inst Oswaldo Cruz. 2007;102(8):919-24.
4. Terenius O, Lindh JM, Eriksson-Gonzales K, Bussiere L, Laugen AT, Bergquist H, et al. Midgut bacterial dynamics in Aedes aegypti. FEMS Microbiol Ecol. 2012;80(3):556-65.
5. Shane JL, Bongio NJ, Favia G, Lampe DJ. Draft Genome Sequence of Asaia sp. Strain SF2.1, an Important Member of the Microbiome of Anopheles Mosquitoes. Genome Announc. 2014;2(1).

6. Chavshin AR, Oshaghi MA, Vatandoost H, Pourmand MR, Raeisi A, Enayati AA, et al. Identification of bacterial microflora in the midgut of the larvae and adult of wild caught Anopheles stephensi: a step toward finding suitable paratransgenesis candidates. Acta Trop. 2012;121(2):129-34.

7. Murakami S, Kanazawa M, Sugishima M, Ogawa A, Ohba T. Relationship between bacterial translocation and isolation of enteric bacteria from extraintestinal organs in slaughtered pigs. J Vet Med Sci. 2011;73(12):1553-60.

8. Gravato-Nobre MJ, Hodgkin J. Caenorhabditis elegans as a model for innate immunity to pathogens. Cell Microbiol. 2005;7(6):741-51.

9. Skriwan C, Fajardo M, Hagele S, Horn M, Wagner M, Michel R, et al. Various bacterial pathogens and symbionts infect the amoeba Dictyostelium discoideum. Int J Med Microbiol. 2002;291(8):615-24.

10. Mateos M, Castrezana SJ, Nankivell BJ, Estes AM, Markow TA, Moran NA. Heritable endosymbionts of Drosophila. Genetics. 2006;174(1):363-76.

11. Bertelli C, Greub G. Lateral gene exchanges shape the genomes of amoeba-resisting microorganisms. Front Cell Infect Microbiol. 2012;2:110.

12. Sun HY, Noe J, Barber J, Coyne RS, Cassidy-Hanley D, Clark TG, et al. Endosymbiotic bacteria in the parasitic ciliate Ichthyophthirius multifiliis. Appl Environ Microbiol. 2009;75(23):7445-52.

13. Beier CL, Horn M, Michel R, Schweikert M, Gortz HD, Wagner M. The genus Caedibacter comprises endosymbionts of Paramecium spp. related to the Rickettsiales (Alphaproteobacteria) and to Francisella tularensis (Gammaproteobacteria). Appl Environ Microbiol. 2002;68(12):6043-50.

14. Fujishima M, Kodama Y. Endosymbionts in paramecium. Eur J Protistol. 2012;48(2):124-37.

15. Berrilli F, Di Cave D, Cavallero S, D'Amelio S. Interactions between parasites and microbial communities in the human gut. Front Cell Infect Microbiol. 2012;2:141.

16. Nakajima T, Sano A, Matsuoka H. Auto-/heterotrophic endosymbiosis evolves in a mature stage of ecosystem development in a microcosm composed of an alga, a bacterium and a ciliate. Biosystems. 2009;96(2):127-35.

17. Siegmund L, Burmester A, Fischer MS, Wostemeyer J. A model for endosymbiosis: interaction between Tetrahymena pyriformis and Escherichia coli. Eur J Protistol. 2013;49(4):552-63.
18. Fichorova RN, Buck OR, Yamamoto HS, Fashemi T, Dawood HY, Fashemi B, et al. The villain team-up or how Trichomonas vaginalis and bacterial vaginosis alter innate immunity in concert. Sex Transm Infect. 2013;89(6):460-6.
19. Smutna T, Goncalves VL, Saraiva LM, Tachezy J, Teixeira M, Hrdy I. Flavodiiron protein from Trichomonas vaginalis hydrogenosomes: the terminal oxygen reductase. Eukaryot Cell. 2009;8(1):47-55.
20. Alves JM, Klein CC, da Silva FM, Costa-Martins AG, Serrano MG, Buck GA, et al. Endosymbiosis in trypanosomatids: the genomic cooperation between bacterium and host in the synthesis of essential amino acids is heavily influenced by multiple horizontal gene transfers. BMC Evol Biol. 2013;13:190.
21. Alves JM, Voegtly L, Matveyev AV, Lara AM, da Silva FM, Serrano MG, et al. Identification and phylogenetic analysis of heme synthesis genes in trypanosomatids and their bacterial endosymbionts. PLoS One. 2011;6(8):e23518.
