# Supplementary material for: Horizontal transfer of β-carbonic anhydrase genes from prokaryotes to protozoans, insects, and nematodes
Source: Parasit Vectors. 2016 Mar 16;9:152. doi: 10.1186/s13071-016-1415-7 (PMC4793742; doi:10.1186/s13071-016-1415-7)
Supplement: Additional file 2: — Prediction of subcellular localization of in vitro-approved prokaryotic endosymbionts and protozoan β-CA protein sequences. (PDF 394 kb) [file 13071_2016_1415_MOESM2_ESM.pdf]

Additional file 2. Prediction of subcellular localization of *in vitro*-approved prokaryotic endosymbionts and protozoan β-CA protein sequences.

Species name	Taxonomic domain	Entry ID	SP*	mTP*	O*	Result*
Afipia felis	Bacteria	K8NQ88	-	+	-	M
Bradyrhizobium japonicum	Bacteria	G7D846	-	-	+	O
Cesiribacter andamanensis	Bacteria	M7MX87	-	-	+	O
Colwellia psychrerythraea	Bacteria	Q47YG3	-	-	+	O
Corallococcus coralloides	Bacteria	H8MJ17	-	+	-	M
Leptospira kirschneri	Bacteria	M6X652	-	+	-	M
Magnetospirillum magneticum	Bacteria	Q2VZD0	-	-	+	O
Selenomonas ruminantium	Bacteria	I0GLW8	-	-	+	O
Veillonella spp.	Bacteria	F9N508	-	-	+	O
Vesicomyosocius okutanii	Bacteria	A5CVM8	-	-	+	O
Acanthamoeba castellanii	Eukaryota	L8GR38	-	+	-	M
Acanthamoeba castellanii	Eukaryota	L8H861	+	-	-	S
Acanthamoeba castellanii	Eukaryota	L8GLS7	-	-	+	O
Angomonas deanei	Eukaryota	S9WXX9	-	-	+	O
Dictyostelium discoideum	Eukaryota	Q555A3	-	-	+	O
Dictyostelium discoideum	Eukaryota	Q55BU2	-	-	+	O
Dictyostelium discoideum	Eukaryota	Q94473	-	-	+	O
Dictyostelium purpureum	Eukaryota	F0Z7L1	-	-	+	O

Dictyostelium fasciculatum	Eukaryota	F4PL43	-	-	+	O
Entamoeba dispar	Eukaryota	B0E7M0	-	-	+	O
Entamoeba histolytica	Eukaryota	C4LXK3	-	-	+	O
Entamoeba nuttalli	Eukaryota	K2GQM0	-	-	+	O
Ichthyophthirius multifiliis	Eukaryota	G0QYZ1	-	-	+	O
Ichthyophthirius multifiliis	Eukaryota	G0QPN9	-	-	+	O
Leishmania braziliensis	Eukaryota	A4H4M7	-	+	-	M
Leishmania donovani	Eukaryota	E9B8S3	-	-	+	O
Leishmania guyanensis	Eukaryota	S0CTX5	-	+	-	M
Leishmania infantum	Eukaryota	A4HSV2	-	-	+	O
Leishmania major	Eukaryota	Q4QJ17	-	-	+	O
Leishmania mexicana	Eukaryota	E9AKU0	-	-	+	O
Naegleria gruberi	Eukaryota	D2W492	-	-	+	O
Naegleria gruberi	Eukaryota	D2W1R2	-	-	+	O
Naegleria gruberi	Eukaryota	D2W4H2	-	-	+	O
Paramecium tetraurelia	Eukaryota	A0BD61	-	-	+	O
Paramecium tetraurelia	Eukaryota	A0E8J0	-	-	+	O
Paramecium tetraurelia	Eukaryota	A0CEX6	-	-	+	O
Paramecium tetraurelia	Eukaryota	A0BDB1	-	-	+	O
Paramecium tetraurelia	Eukaryota	A0C922	-	-	+	O
Strigomonas culicis	Eukaryota	S9TM82	-	+	-	M

Tetrahymena thermophila	Eukaryota	Q22U21	-	-	+	O
Tetrahymena thermophila	Eukaryota	Q22U16	-	-	+	O
Tetrahymena thermophila	Eukaryota	I7MDL7	-	+	-	M
Tetrahymena thermophila	Eukaryota	I7LWM1	-	-	+	O
Tetrahymena thermophila	Eukaryota	I7M0M0	-	-	+	O
Tetrahymena thermophila	Eukaryota	I7MD92	-	-	+	O
Tetrahymena thermophila	Eukaryota	I7M748	-	-	+	O
Tetrahymena thermophila	Eukaryota	Q23AV1	-	-	+	O
Trichomonas vaginalis	Eukaryota	A2ENQ8	-	-	+	O
Trichomonas vaginalis	Eukaryota	A2DLG4	-	-	+	O
Ascaris suum	Eukaryota	F1LE18	-	-	+	O
Schistosoma mansoni	Eukaryota	G4V6B2	-	-	+	O
Caenorhabditis elegans	Eukaryota	Q22460	-	-	+	O
Anopheles gambiae	Eukaryota	Q5TU56	-	+	-	M
Aedes aegypti	Eukaryota	Q17N64	-	+	-	M
Drosophila melanogaster	Eukaryota	Q9VHJ5	-	+	-	M

***Abbreviations:**

M: Mitochondrial, **SP:** Signal peptide (secretory pathway), **mTP:** (mitochondrial targeting peptide), and **O:** other cellular localization.
