# Supplementary material for: Horizontal transfer of β-carbonic anhydrase genes from prokaryotes to protozoans, insects, and nematodes
Source: Parasit Vectors. 2016 Mar 16;9:152. doi: 10.1186/s13071-016-1415-7 (PMC4793742; doi:10.1186/s13071-016-1415-7)
Supplement: Additional file 3: — Bacterial MGEs containing β-CA, transposase, integrase, resolvase, and CCP coding sequences. (PDF 338 kb) [file 13071_2016_1415_MOESM3_ESM.pdf]

Additional file 3. Bacterial MGEs containing β-CA, transposase, integrase, resolvase, and CCP coding sequences.

Bacterial species	MGE name	MGE proteins	Coding IDs from ACLAME	Coding IDs from GenBank
Acaryochloris marina	**NCBI:** pREB1	β-CA	-	ABW31395.1
		Transposase	-	ABW31524.1
		Integrase	-	ABW31698.1
		Resolvase	-	-
		Conjugative complex proteins	-	-
***Azospirillum* spp.**	**NCBI:** pAB510c	β-CA	-	YP_003452337.1
		Transposase	-	YP_003451879.1
		Integrase	-	YP_003452221.1
		Resolvase	-	-
		Conjugative complex proteins	-	-
Bacillus bombysepticus	**NCBI:** pBb	β-CA	-	AHX21627.1
		Transposase	-	AHX21699.1
		Integrase	-	AHX21668.1
		Resolvase	-	AHX21737.1
		Conjugative complex proteins	-	AHX21674.1
Bacillus cereus	**NCBI:** pAH1134_566	β-CA	-	WP_000926479.1
		Transposase	-	WP_001988148.1
		Integrase	-	WP_000846668.1
		Resolvase	-	WP_001154652.1
		Conjugative complex proteins	-	WP_000450205.1
Bacillus thuringiensis	**NCBI:** pBMB0231	β-CA	-	YP_008822632.1
		Transposase	-	YP_008822571.1
		Integrase	-	YP_008822639.1
		Resolvase	-	YP_008822718.1
		Conjugative complex proteins	-	-
Bacillus thuringiensis **(serovar *Kurstaki*, str. YBT-1520)**	**NCBI:** pBMB400	β-CA	-	AIM34891.1
		Transposase	-	AIM34842.1
		Integrase	-	AIM34843.1
		Resolvase	-	AIM34919.1
		Conjugative complex proteins	-	AIM34845.1

***Burkholderia* spp.**	**NCBI:** p1	β-CA<br>Transposase<br>Integrase<br>Resolvase<br>Conjugative complex proteins	-<br>-<br>-<br>-<br>-	BAO91749.1<br>-<br>BAO92497.1<br>YP_001984281.1<br>-
Burkholderia phymatum	**NCBI:** pBPHY01	β-CA<br>Transposase<br>Integrase<br>Resolvase<br>Conjugative complex proteins	-<br>-<br>-<br>-<br>-	ACC75223.1<br>ACC74803.1<br>ACC74635.1<br>ACC75957.1<br>-
Eubacterium eligens **(ATCC 27750)**	**NCBI:** Unnamed	β-CA<br>Transposase<br>Integrase<br>Resolvase<br>Conjugative complex proteins	-<br>-<br>-<br>-<br>-	YP_002935894.1<br>YP_002935430.1<br>YP_002935625.1<br>-<br>-
Halopiger xanaduensis	**NCBI:** pHALXA01	β-CA<br>Transposase<br>Integrase<br>Resolvase<br>Conjugative complex proteins	-<br>-<br>-<br>-<br>-	AEH39227.1<br>AEH38917.1<br>AEH39015.1<br>-<br>-
Methylobacterium oryzae	**NCBI:** pMOC3	β-CA<br>Transposase<br>Integrase<br>Resolvase<br>Conjugative complex proteins	-<br>-<br>-<br>-<br>-	AGO88419.1<br>-<br>-<br>AGO88421.1<br>AGO88429.1
Methylobacterium radiotolerans	**NCBI:** pMRAD01	β-CA<br>Transposase<br>Integrase<br>Resolvase<br>Conjugative complex proteins	-<br>-<br>-<br>-<br>-	ACB28042.1<br>ACB27983.1<br>ACB27982.1<br>-<br>ACB28029.1
***Nitrosomonas* spp.**	**NCBI:** pNAL21201	β-CA<br>Transposase<br>Integrase<br>Resolvase<br>Conjugative complex proteins	-<br>-<br>-<br>-<br>-	ADZ27963.1<br>ADZ27964.1<br>ADZ28028.1<br>-<br>ADZ27976.1

***Pantoea* spp.**	**NCBI:** pPAT9B03	β-CA<br>Transposase<br>Integrase<br>Resolvase<br>Conjugative complex proteins	-<br>-<br>-<br>-<br>-	YP_004118978.1<br>-<br>YP_004119028.1<br>-<br>-
Ralstonia solanacearum**<br>(str. GMI1000)**	**ACLAME:** mge:570<br>**NCBI:** pGMI1000MP	β-CA<br>Transposase<br>Integrase<br>Resolvase<br>Conjugative complex proteins	protein:plasmid:21126<br>protein:plasmid:21140<br>protein:plasmid:21916<br>-<br>-	NP_521673.1<br>NP_521687.1<br>NP_522463.1<br>-<br>-
Rhizobium etli**<br>(str. CFN 42)**	**ACLAME:** mge:572<br>**NCBI:** p42d	β-CA<br>Transposase<br>Integrase<br>Resolvase<br>Conjugative complex proteins	protein:plasmid:22829<br>protein:plasmid:22747<br>protein:plasmid:22785<br>protein:plasmid:23020<br>protein:plasmid:22854	NP_659846.2<br>AAM48297.1<br>NP_659803.1<br>NP_660035.1<br>NP_659871.1
***Rhizobium* spp.**	**NCBI:** pLPU83d	β-CA<br>Transposase<br>Integrase<br>Resolvase<br>Conjugative complex proteins	-<br>-<br>-<br>-<br>-	CDM62033.1<br>CDM61688.1<br>CDM61764.1<br>-<br>-
Rhizobium etli	**NCBI:** pB	β-CA<br>Transposase<br>Integrase<br>Resolvase<br>Conjugative complex proteins	-<br>-<br>-<br>-<br>-	YP_001984505.1<br>YP_001984279.1<br>YP_001984280.1<br>YP_001984281.1<br>YP_001984445.1
Rhizobium grahamii	**NCBI:** pRg502a	β-CA<br>Transposase<br>Integrase<br>Resolvase<br>Conjugative complex proteins	-<br>-<br>-<br>-<br>-	WP_016558599.1<br>WP_016558407.1<br>WP_016558516.1<br>WP_016558625.1<br>WP_016558632.1
Rhodococcus jostii**<br>(str. RHA1)**	**ACLAME:** mge:814<br>**NCBI:** pRHL1	β-CA<br>Transposase<br>Integrase<br>Resolvase<br>Conjugative complex proteins	protein:plasmid:111105<br>protein:plasmid:111103<br>protein:plasmid:111217<br>protein:plasmid:111613<br>-	YP_707498.1<br>YP_707496.1<br>YP_707218.1<br>YP_707727.1<br>-

Salmonella typhimurium<br>**(str. LT2)**	**ACLAME:** mge:596<br>**NCBI:** pSLT	β-CA<br>Transposase<br>Integrase<br>Resolvase<br>Conjugative complex proteins	protein:plasmid:24850<br>protein:plasmid:24840<br>protein:plasmid:24846<br>protein:plasmid:24836<br>protein:plasmid:24883	NP_490536.1<br>NP_490526.1<br>NP_490532.1<br>NP_490521.1<br>NP_490568.1
Salmonella enterica<br>**(serovar *Choleraesuis*)**	**ACLAME:** mge:586<br>**NCBI:** pKDSC50	β-CA<br>Transposase<br>Integrase<br>Resolvase<br>Conjugative complex proteins	protein:plasmid:24077<br>protein:plasmid:24034<br>-<br>-<br>-	NP_073268.1<br>NP_073225.1<br>-<br>-<br>-
Salmonella enterica	**ACLAME:** mge:969<br>**NCBI:** pOU1113	β-CA<br>Transposase<br>Integrase<br>Resolvase<br>Conjugative complex proteins	protein:plasmid:156785<br>protein:plasmid:156789<br>protein:plasmid:156786<br>protein:plasmid:156759<br>protein:plasmid:156802	YP_271809.1<br>YP_271735.1<br>YP_271811.1<br>YP_271739.1<br>YP_271767.1
Salmonella enterica<br>**(serovar *Choleraesuis*, str. SC-B67)**	**ACLAME:** mge:1105<br>**NCBI:** pSCV50	β-CA<br>Transposase<br>Integrase<br>Resolvase<br>Conjugative complex proteins	protein:plasmid:151454<br>protein:plasmid:151440<br>protein:plasmid:151447<br>protein:plasmid:151480<br>-	YP_209302.1<br>YP_209255.1<br>YP_209303.1<br>YP_209268.1<br>-
Salmonella enterica<br>**(serovar *Typhimurium*, str. U288)**	**NCBI:** pSTU288-1	β-CA<br>Transposase<br>Integrase<br>Resolvase<br>Conjugative complex proteins	-<br>-<br>-<br>-<br>-	AGK12338.1<br>AGK12397.1<br>AGK12231.1<br>AGK12231.1<br>AGK12297.1
Salmonella enterica<br>**(serovar *Typhimurium*, str. 138736)**	**NCBI:** Unnamed	β-CA<br>Transposase<br>Integrase<br>Resolvase<br>Conjugative complex proteins	-<br>-<br>-<br>-<br>-	AHX80301.1<br>AHX80290.1<br>AHX80299.1<br>AHX80284.1<br>AHX80330.1
Salmonella enterica<br>**(serovar *Gallinarum*, str. SG9)**	**NCBI:** Unnamed	β-CA<br>Transposase<br>Integrase<br>Resolvase	-<br>-<br>-<br>-	WP_001541566.1<br>WP_031606504.1<br>WP_000098784.1<br>WP_000082170.1

		Conjugative complex proteins	-	WP_000129019.1
Salmonella enterica	**NCBI:** p798_93	β-CA	-	YP_005412542.1
**(serovar *Typhimurium*, str. 798)**		Transposase	-	YP_005412530.1
		Integrase	-	YP_005412537.1
		Resolvase	-	YP_005412523.1
		Conjugative complex proteins	-	YP_005412569.1
Salmonella enterica	**NCBI:** pSD_77	β-CA	-	YP_006954912.1
**(serovar *Dublin*)**		Transposase	-	YP_006954895.1
		Integrase	-	YP_006954910.1
		Resolvase	-	YP_006954890.1
		Conjugative complex proteins	-	YP_006954969.1
Salmonella enterica	**NCBI:** p3246_74	β-CA	-	WP_001541566.1
**(serovar *Dublin*, str. SD3246)**		Transposase	-	WP_000064919.1
		Integrase	-	WP_000098783.1
		Resolvase	-	WP_000082169.1
		Conjugative complex proteins	-	WP_000129019.1
Salmonella enterica	**NCBI:** Unnamed	β-CA	-	AHW04041.1
**(serovar *Enteritidis*, str. Durban)**		Transposase	-	AHW03979.1
		Integrase	-	AHW04043.1
		Resolvase	-	AHW03986.1
		Conjugative complex proteins	-	AHW04013.1
Salmonella enterica	**NCBI:** pOU7519	β-CA	-	ABX56772.1
**(serovar *Choleraesuis*, str. OU7519)**		Transposase: -	-	ABX56727.1
		Integrase	-	ABX56741.1
		Resolvase	-	ABX56741.1
		Conjugative complex proteins	-	ABX56759.1
Salmonella enterica	**NCBI:** pOU1115	β-CA	-	ACA51134.1
**(serovar *Dublin*)**		Transposase	-	ACA51210.1
		Integrase	-	ACA51132.1
		Resolvase	-	ACA51205.1
		Conjugative complex proteins	-	ACA51211.1
Salmonella enterica	**NCBI:** pYT1	β-CA	-	BAJ15344.1
**(serovar *Typhimurium*)**		Transposase	-	BAJ15296.1
		Integrase	-	BAJ15315.1
		Resolvase	-	BAJ15319.1

		Conjugative complex proteins	-	BAJ15371.1
Salmonella enterica<br>**(serovar *Typhimurium*)**	**NCBI:** pDT104	β-CA<br>Transposase<br>Integrase<br>Resolvase<br>Conjugative complex proteins	-<br>-<br>-<br>-<br>-	CCW77330.1<br>CCW77319.1<br>CCW77305.1<br>CCW77313.1<br>CCW77360.1
Salmonella enterica<br>**(serovar *Typhimurium*, str. L-3553)**	**NCBI:** pST3553	β-CA<br>Transposase<br>Integrase<br>Resolvase<br>Conjugative complex proteins	-<br>-<br>-<br>-<br>-	BAP10700.1<br>BAP10624.1<br>BAP10644.1<br>BAP10666.1<br>BAP10735.1
Salmonella enterica<br>**(serovar *Typhimurium*, str. SL1344)**	**NCBI:** pSLT_SL1344	β-CA<br>Transposase<br>Integrase<br>Resolvase<br>Conjugative complex proteins	-<br>-<br>-<br>-<br>-	CCF76766.1<br>CCF76762.1<br>CCF76768.1<br>CCF76767.1<br>CCF76712.1
Salmonella enterica<br>**(serovar *Typhimurium*, str. T000240)**	**NCBI:** pSTMDT12_L	β-CA<br>Transposase<br>Integrase<br>Resolvase<br>Conjugative complex proteins	-<br>-<br>-<br>-<br>-	BAJ39737.1<br>BAJ39704.1<br>BAJ39697.1<br>BAJ39688.1<br>BAJ39769.1
***Silicibacter* spp.**<br>**(str. TM1040)**	**ACLAME:** mge:1094<br>**NCBI:** TM1040 mega plasmid	β-CA<br>Transposase<br>Integrase<br>Resolvase<br>Conjugative complex proteins	protein:plasmid:134531<br>protein:plasmid:134438<br>protein:plasmid:134429<br>-<br>-	YP_611691.1<br>YP_611363.1<br>YP_611354.1<br>-<br>-
***Streptomyces* spp.**	**NCBI:** pFRL6	β-CA<br>Transposase<br>Integrase<br>Resolvase<br>Conjugative complex proteins	-<br>-<br>-<br>-<br>-	AHE40322.1<br>AHE40263.1<br>AHE40253.1<br>AIM34919.1<br>AIM34845.1
