# Supplementary material for: Horizontal transfer of β-carbonic anhydrase genes from prokaryotes to protozoans, insects, and nematodes
Source: Parasit Vectors. 2016 Mar 16;9:152. doi: 10.1186/s13071-016-1415-7 (PMC4793742; doi:10.1186/s13071-016-1415-7)
Supplement: Additional file 4: — Genomic location of β-CA gene sequences from protozoan, insect, and nematode species. (PDF 363 kb) [file 13071_2016_1415_MOESM4_ESM.pdf]

Additional file 4. Genomic location of *β-CA* gene sequences from protozoan and metazoan species.

Species name	Entry ID	Gene name	Exon count	Chromosome No.	Genomic sequence
Acanthamoeba castellanii	L8GR38	ACA1_164750	8	Unknown	264,624-266,036
	L8H861	ACA1_278940	4	Unknown	280,801-281,829
	L8GLS7	ACA1_365670	7	Unknown	289,130-290,585
Dictyostelium spp.	Q555A3	DDB_G0274643	2	2	4,531,583-4,532,438
	Q55BU2*	cahA	1	1	4,712,273-4,713,103
	Q94473*	cahA	1	1	4,712,273-4,713,103
	F0Z7L1	DICPUDRAFT_147072	1	Unknown	83,578-84,354
	F4PL43	DFA_05397	2	Unknown	1,443,296-1,444,125
Entamoeba spp.	B0E7M0	EDI_275880	1	Unknown	31,344-31,910
	C4LXK3	EHI_073380	1	Unknown	17,880-18,446
	K2GQM0	ENU1_204230	1	Unknown	5,440-6,006
Ichthyophthirius multifiliis	G0QYZ1	IMG5_153340	1	Unknown	81,247-82,352
	G0QPN9	IMG5_069900	1	Unknown	37,943-38,641
Leishmania spp.	A4H4M7	LBRM_06_0620	1	6	215,004 - 216,170
	E9B8S3	LDBPK_060630	1	6	218,575-219,495
	A4HSV2	LINJ_06_0630	1	6	216,541-217,461
	Q4QJ17	LMJF_06_0610	1	6	215,136-216,056
	E9AKU0	LMXM_06_0610	1	6	211,291-212,211
Naegleria gruberi	D2W492	NAEGRDRAFT_76222	2	Unknown	10,696-12,198
	D2W1R2	NAEGRDRAFT_75346	1	Unknown	141,879-142,928
	D2W4H2	NAEGRDRAFT_76305	1	Unknown	28,890-29,417
Paramecium tetraurelia	A0BD61	GSPATT00004572001	3	Unknown	601,533-602,458
	A0E8J0	GSPATT00024336001	3	Unknown	10,540-11,572
	A0CEX6	GSPATT00037782001	3	Unknown	66,521-67,437

	A0BDB1<br>A0C922	GSPATT00004622001<br>GSPATT00006595001	3<br>3	Unknown<br>Unknown	676,997-677,921<br>54,594-55,511
Tetrahymena thermophila	Q22U21<br>Q22U16<br>I7MDL7<br>I7LWM1<br>I7M0M0<br>I7MD92<br>I7M748<br>Q23AV1	TTHERM_00263620<br>TTHERM_00263670<br>TTHERM_00373840<br>TTHERM_00558270<br>TTHERM_00374880<br>TTHERM_00541480<br>TTHERM_00374870<br>TTHERM_00654260	1<br>2<br>1<br>4<br>1<br>1<br>1<br>1	Unknown<br>Unknown<br>Unknown<br>Unknown<br>Unknown<br>Unknown<br>Unknown<br>Unknown	608,825-609,616<br>628,697-629,487<br>126,279-127,092<br>243,468-245,325<br>115,394-116,473<br>457,999-458,646<br>117,488-118,991<br>563,482-564,490
Trichomonas vaginalis	A2ENQ8<br>A2DLG4	TVAG_005270<br>TVAG_268150	1<br>1	Unknown<br>Unknown	9,495-10,043<br>151,119-151,673
Aedes aegypti	Q17N64	AAEL000816	2	Unknown	175,436-176,154
Anopheles gambiae	Q5TU56	AgaP_AGAP002992	5	2R	892,475-893,390
Ascaris suum	F1LE18	N/A	1	Unknown	32-811
Caenorhabditis elegans	Q22460	bca-1 gene	7	Chromosome X	23,095-25,694
Drosophila melanogaster	Q9VHJ5	CAHbeta	3	3R	8,980,860-8,982,793
Schistosoma mansoni	G4V6B2	Smp_004070	5	Unknown	5,648,179-5,666,146

*: There is a three nucleotides difference between the sequence from the Sequencing Center and the sequence in GenBank record, which resulted in two amino acid substitutions at two different positions (Entry ID: Q55BU2 and Q94473). This information was obtained from dictyBase database (http://dictybase.org/).
