# Supplementary figures and images for: Horizontal transfer of β-carbonic anhydrase genes from prokaryotes to protozoans, insects, and nematodes
Source: Parasit Vectors. 2016 Mar 16;9:152. doi: 10.1186/s13071-016-1415-7 (PMC4793742; doi:10.1186/s13071-016-1415-7)

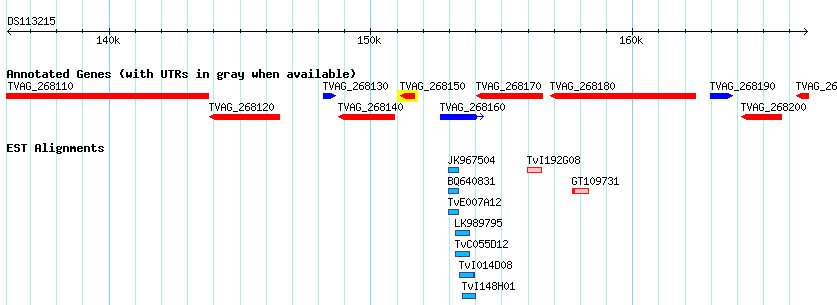

Supplement: Additional file 5: — Location of β-CA gene sequence (TVAG_268150) in T. vaginalis. This gene (Entry ID: A2DLG4) has been located on the linear main genomic DNA sequence from 151,119 to 151,673 nt. Analysis revealed that it consists of only one exon (Additional file 4). (TIF 45 kb) [file 13071_2016_1415_MOESM5_ESM.tif]

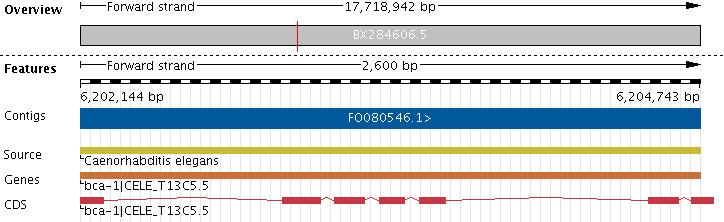

Supplement: Additional file 6: — Location of β-CA gene sequence (bca-1) in C. elegans. This gene (Entry ID: Q22460) has been located on linear main genomic DNA sequence from 23,095 to 25,694 nt. Analysis revealed that it consists of seven exons (Additional file 4). (TIF 132 kb) [file 13071_2016_1415_MOESM6_ESM.tif]
